# Microstructure and Properties of Nanostructured Coating on Ti6Al4V

**DOI:** 10.3390/ma13030708

**Published:** 2020-02-05

**Authors:** Veronika Jordanovová, Monika Losertová, Michal Štencek, Tereza Lukášová, Gražyna Simha Martynková, Pavlína Peikertová

**Affiliations:** 1 Faculty of Materials Science and Technology, VŠB-Technical University of Ostrava, 17. listopadu 2172/15, 708 00 Ostrava, Poruba, Czech Republic; veronika.jordanovova@vsb.cz (V.J.); tereza.lukasova@vsb.cz (T.L.); 2 Nanotechnology Centre, VŠB—Technical University of Ostrava, 17. listopadu 2172/15, 708 00 Ostrava, Poruba, Czech Republic; grazyna.simha@vsb.cz (G.S.M.); pavlina.peikertova@vsb.cz (P.P.); 3 IT4Innovations Centre of Excellence, VŠB—Technical University of Ostrava, 17. listopadu 2172/15, 708 00 Ostrava, Poruba, Czech Republic

**Keywords:** Ti6Al4V, TiO_2_ nanotubes, oxide coating, anodization, microstructure, corrosion, SEM

## Abstract

Implant surface properties of Ti6Al4V alloy that is currently used as a biocompatible material because of a variety of unique properties can be improved by a self-organized TiO_2_ layer. The TiO_2_ nanotubes forming on the titanium-based materials is a relatively recent technology for the surface properties modification and represents pronounced potential in promoting cell adhesion, proliferation, and differentiation that facilitate an implant osseointegration. This work focuses on the influence of surface treatment quality and anodic oxidation parameters on the structure features and properties of TiO_2_ nanotube coatings. The nanotubes were formed on Ti6Al4V alloy substrates by simultaneous surface oxidation and controlled dissolving of an oxide film in the presence of fluorine ions. The anodization process on ground or polished samples was performed at experimental condition of 30 V for 1 h. The selected anodized samples were heat treated for 2 h at 500 °C under flowing argon. All samples were characterized by scanning electron microscopy, X-ray diffraction analysis, and Raman spectroscopy. The corrosion rate in physiological solution reached 0.0043, 0.0182, and 0.0998 mm per year for the samples in polished and not-anodized, as-anodized, and anodized-heat treated conditions, respectively.

## 1. Introduction

Nanomaterials appear currently not only as an important part of science and technology but have become an essential requirement for improving the properties of some common life products. Extremely fast development in nanotechnology is due to the unique properties of nanomaterials in a wide range of applications. Nowadays, great attention is paid to TiO_2_ nanotubes (NT) [1,2,3], which have a huge potential for use in gas sensors, solar cells, photocatalytic, and also in medical applications. Current implants in biomedicine that are made from 316L stainless steel or Ti6Al4V alloy show an ever-increasing degree of negative reactions in the corrosive environment of the human body which subsequently affect not only the viability of the implanted material, but also causes serious complications of the patient’s health. Such problems may include allergic or inflammatory reactions associated with medical devices, such as orthopedic, traumatology, surgery, or dental implants. Indeed, excellent biocompatibility of implants with bone tissue with restrictions due to the possibility of bacterial infections persists as the main problems in biomedicine [4].

The Ti6Al4V alloy that is currently used as a biocompatible material because of a variety of unique properties can be corroded by a contact with corrosive body fluids, even if the surface is protected by an oxide layer [5]. On the other side, a modification of the surface structure due to self-organized TiO_2_ NT can facilitate implant osseointegration [6].

Optimization of corrosive properties of implant surfaces can be achieved by various methods such as chemical (CVD) or physical (PVD) vapor deposition, thermal or electrochemical treatment. Nevertheless, an anodization (or electrolytic oxidation) process is a very common method of the surface treatment and is often used in the case of titanium or aluminum and its alloys. Relatively last modifications of process parameters allow the preparation of uniform nanostructured coatings. Moreover, the anodic oxidation method enabling the growth of highly ordered TiO_2_ NT is a very simple, inexpensive, flexible, and versatile method, and limits or eliminates the undesirable effects of corrosion or inflammation mentioned above.

Several methods of the anodization of Ti or Ti6Al4V substrates have been performed in aqueous, non-aqueous, or organic electrolytes, resulting in required morphological and geometric parameters of NT [1]. Various process parameters such as voltage, type, composition, and temperature of the electrolyte significantly affect the physical properties and morphology of TiO_2_ NT. Less attention is paid to the effects of stress produced by inappropriate voltage parameters. According to [3], the formation of TiO_2_ NT at a voltage higher than 80 V is almost impossible. Indeed, most recent results in [7,8,9] concerning anodization process were evaluated for voltage parameters from 10–75 V.

Another important effect that can modify the NT layer properties is the feature of the substrate surface. In the case of Ti6Al4V, alloy character of anodized layers is considerably influenced by microstructure of the alloy, and thus by morphology of phases α (hexagonal closed package structure-HCP) and β (body centered cubic structure-BCC) [10] that reacts differentially [11] in chemical solutions.

As the NT applied on the surface of skeletal/dental implants are mainly intended to prevent implant rejection and consequently prevent life-threatening secondary surgeries [4], the impact of the structural and morphological aspects of TiO_2_ NT on their application as implant coating is significant. It has been already remarked using in vitro studies that the morphology and composition of TiO_2_ NT influence biocompatibility [8,12], thereby showing bioactivity of bone cells on titanium implant surfaces. However, further studies are needed to determine the anodizing parameters as well as the microstructure effect resulting in internal stresses in NT coatings.

In this paper, the effect of the surface conditions and the corrosive properties of the substrates without or with TiO_2_ NT coatings produced by means of the anodization process was studied. The morphology and structure of the anodized coatings before and after heat treatment was analyzed and discussed in the context of the substrate microstructure.

## 2. Materials and Methods

### 2.1. Materials

The specimens of circular cross section of 20 mm in diameter and 5 mm in depth were prepared from rods of biocompatible Ti6Al4V alloy (Grade 5), supplied by MEDIN a.s., Czech Republic. The material used is consistent with CSN EN ISO 5832-3, ASTM F620-11, and ASTM F1472-14.

Before anodization process, the sample surfaces were treated by mechanical grinding on SiC abrasive papers (Grit 600, 1000, and 2500) and polishing on a woven DP-Mol cloth in 1 µm, 0.3 µm, and 0.05 µm alumina suspensions (metallographic material supplied by STRUERS, GmbH, Roztoky u Prahy, Czech Republic).

### 2.2. Synthesis of TiO_2_ Nanotubes

Nanotubular layers were prepared on the sample surfaces using the electrochemical anodization method. The ground or polished discs were immersed in an electrolytic solution composed of ethylene glycol (EG), distilled water, and ammonium fluoride NH_4_F in a ratio of 89:10:1 (chemicals supplied by MERCK, Darmstadt, Germany) and anodized for 60 min at 23 °C at 30 V voltage using DC Power Supply MCS-3204 MANSON device (Manson, Hong Kong). A two-electrode electrochemical cell with Ti6Al4V sample as working electrode (anode) and platinum mesh as counter electrode (cathode) was used for the anodization process. After anodization, the samples were rinsed with distilled water and dried.

Selected anodized samples were heat treated (HT) in LINN HT1800 (Eschenfelden, Germany) furnace under flowing argon with the regime as follows: heating rate of 16.7 °C/minute, holding time of 120 min at 500 °C, followed by slow furnace cooling.

### 2.3. Microstructure and Surface Characterization

Prior to anodization, the surface microstructure of the Ti6Al4V samples were investigated by optical microscopy (OM), scanning electron microscopy (SEM), and X-ray diffraction analysis (XRD).

For microstructure study, the Ti6Al4V discs were ground on SiC papers up to Grit 2000 and polished on a woven DP-Mol cloth in 1 µm and 0.3 µm alumina suspension. The Kroll’s etching solution containing 6 mL of nitric acid, 2 mL of hydrofluoric acid and 92 mL of distilled water (chemicals supplied by MERCK, Darmstadt, Germany) was used to reveal the microstructure. The microstructure observation was performed by means of a Lab-robot Top-Eye^TM^ LT microscope (Schoondijke, The Netherlands).

Surface morphology of anodizing surfaces before and after heat treatment at 500 °C was analyzed using the JEOL JSM-6490LV scanning electron microscope (SEM) (JEOL, Tokyo, Japan) equipped with an Oxford INCA x-act energy dispersion spectral (EDS) analyzer. Photo-documentation of the surface study was carried out by mode of secondary electrons (SEI) with an accelerating voltage of 20 kV.

The structure and phase identification of the anodized sample before and after heat treatment was evaluated by X-ray diffraction (XRD) analysis using Ultima IV (Rigaku, Japan) diffractometer with following measurement conditions: Bragg-Brentano configuration and reflection mode without rotation, scan axis 2 theta/theta, acceleration current 40 mA, excitation voltage 40 kV, K beta filter, incident slit 2/3, and receiving slit 0.6 mm with scintillation counter. Recording was performed in the range of 5–80° 2θ CuKα in a step width 0.05° and scan speed 4 degree/min, which was subsequently adjusted for the purpose of evaluation to a range of 20-80° 2θ. For the phase’s evaluation, ICDD JCPDS database PDF-2/2011 was used.

The TiO_2_ NT structure was determined using a XploRA ™ confocal Raman microscope (HORIBA Jobin Yvon, France) with included Olympus BX41/51 optical microscope equipped by 50x magnification lens. The measurement was realized with laser wavelength of 785 nm (20–25 mW, class 3B). The spectra were scanned ten times for 10 s with a 1200 scratches/mm grid. The device is fully automated with the LabSpec computer software that allowed all adjustment of the spectra.

### 2.4. Corrosion Tests

The electrochemical behavior was compared for Ti6Al4V samples in following conditions of the surface: ground or polished (not-anodized), as-anodized, and anodized-heat treated, in order to determine the effect of each preparation step on the corrosion rate. Three-electrode cell was formed of the sample as working electrode, the platinum grid as counter electrode and the AgCl/3M KCl electrode (METROHM, USA) as reference electrode. An infusion solution of 0.9% sodium chloride (Braun) containing Na^+^ and Cl^−^ ions served as electrolyte. Before the corrosion test, the solution was warmed and maintained at the temperature of 37 °C. The corrosion test measurement ranged from −150 to 1000 mV and was carried out on the Autolab PGSTAT128N (METROHM, USA) potentiostat/galvanostat equipped by Nova 1.10 Autolab program. The corrosion behavior of the Ti6Al4V samples was evaluated using the Voltamper characteristics and Tafel extrapolation method.

## 3. Results and Discussion

### 3.1. Microstructure Before and After Anodization

**Figure 1:**
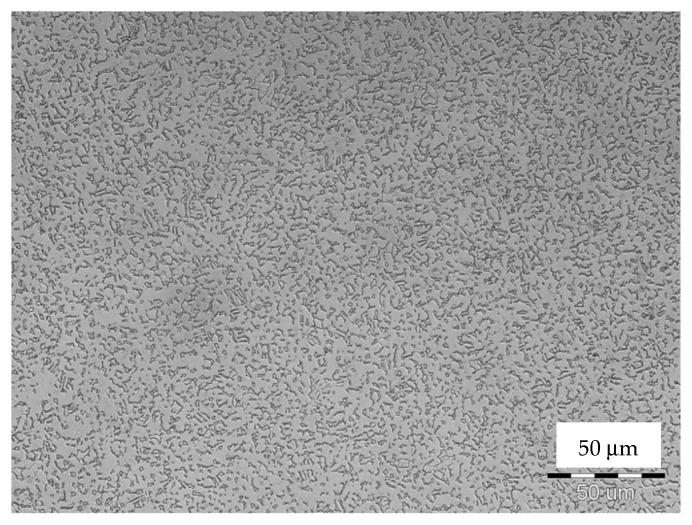
Optical microscopy (OM) microstructure image of etched Ti6Al4V specimen composed of two phases: light α and dark transformed β (etched).

In order to study the morphology of as-anodized and anodized-heat-treated TiO_2_ NT coatings, the microstructure of the substrate sample was determined. The bimodal morphology of the Ti6Al4V alloy that is shown in Figure 1 is formed of equiaxed α-grains and transformed β phase, as it was analyzed in [13,14]. Grains of α-phase are observed as light areas unlike dark transformed β composed of (α+β) lamellae. As an etching attack currently reveal α and β phases differentially, it can be predicted that both phases react differentially also in anodization EG electrolyte containing fluoride ions (NH_4_F).

**Figure 2:**
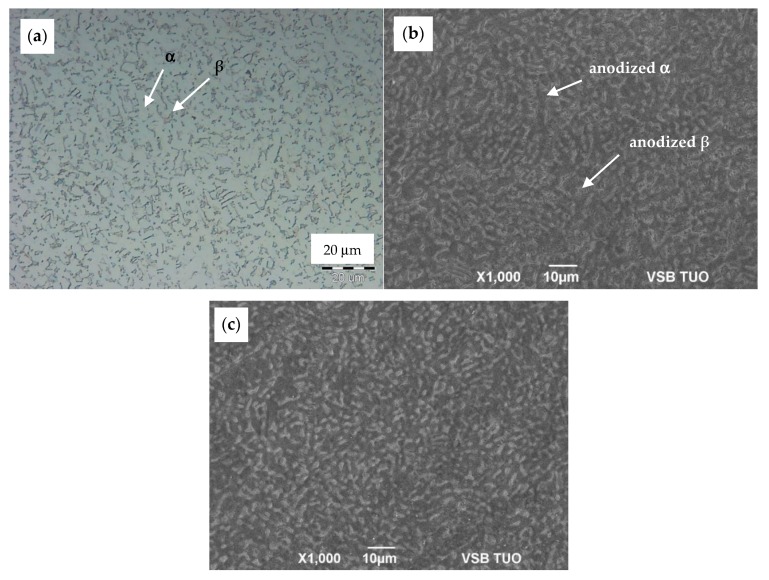
Comparison of: (**a**) OM microstructure of the substrate surface, (**b**) SEM SEI morphology of the as-anodized sample, and (**c**) SEM SEI morphology of the anodized-heat treated sample.

The morphology of as-anodized and anodized-heat-treated samples was observed only by SEM in SEI (secondary electron image) mode. Unlike in optical microscopy (OM) Figure 2a, coloration of both phases in SEM SEI images appears quite conversely: α-phase is observed as dark areas and transformed β-phase as light areas, as seen in Figure 2b. The comparison of the SEM surface morphology of as-anodized and anodized-heat treated samples is presented in Figure 2b,c that revealed the obvious dependence of NT growing on initial α + β microstructure. Appearance of the NT surface after annealing seems to be unchanged.

**Figure 3:**
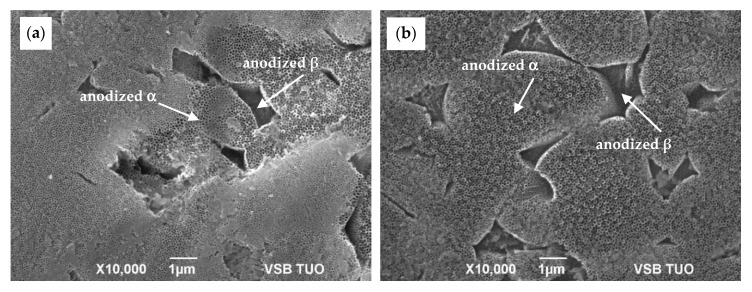
SEM SEI morphology of the anodized surfaces (**a**) ground and (**b**) polished.

Figure 3 shows the regions of transformed β-phase covered by NT, whose features differ considerably from that over the α-phase areas. The non-uniform appearance of the NT layer is caused by the different electrochemical reactions of both phases resulting from their different chemical composition and crystalline structure. Differences between α- and β-phases in Ti content [14] play an important role in the growth of the oxide layer in the EG electrolyte at higher potentials applied [7,8,9,10]. Moreover, diffusion coefficient of Ti atoms varies for both phases, so the bimodal α+β microstructure significantly affects the regularity and homogeneity of the NT surface layers. The rate of oxide layer growth at anodization should be faster for β-phase, thanks to quicker Ti diffusivity into the BCC structure but controversial process of the TiO_2_ dissolution during NT growth proceeds preferentially over the β-regions due to content of vanadium as a β-stabilizing element [15,16]. Thus, the bimodal structure of the Ti6Al4V substrate surface resulted in two distinct NT morphologies.

The comparison of the influence of surface treatment quality on the feature of the NT layer without subsequent heat treatment is presented on the SEM micrograph in Figure 3. The structure of the NT layer prepared on the ground surface is shown in Figure 3a. This image displays a much more irregular roughness of the NT coating unlike the uniform NT distribution for the polished surface, as it is seen in Figure 3b.

The NT growth on a smooth polished surface requires more energy to produce nuclei of NT and subsequent building up in the same crystallographic orientation as the substrate surface. Conversely, surface irregularities caused by grinding lead to easier formation of NT nuclei, but their growth produced is more disorderly. Thus, the NT layer on the ground surface may appear to be more dense as there is more intensive contact between the growing walls of adjacent NT, unlike the smooth surface, with spare distribution and more irregular height of NT. Some parts of the ground substrate is covered by compact oxide layer.

**Figure 4:**
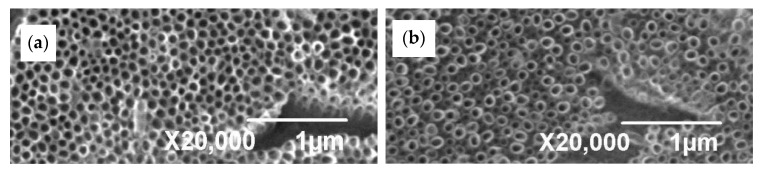
Details of SEM SEI images of the anodized surfaces (**a**) ground and (**b**) polished.

**Figure 5:**
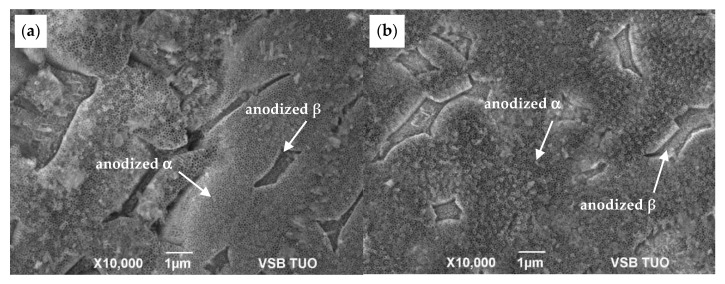
SEM SEI images of the surfaces anodized-heat treated: (**a**) ground and (**b**) polished.

As detailed in Figure 4 and in Table 1 the polished surface allowed growth of the NT having larger outer diameter and wall thickness. As in the case of as-anodized surfaces, the same tendency was noted for annealed state of both ground and polished surfaces, the coating feature remained significantly affected by the surface treatment before anodization (Figure 5).

Several studies [1,3,6,15] confirmed that the as-anodized TiO_2_ nanotubes are in amorphous state. However, when they are heat-treated at higher temperatures, the NTs crystallize to anatase phase or a mix of anatase and rutile phases. The heat treatment activates sintering processes that are accelerated by the large specific surface and the amorphous character of the NT. The presence of the residual amount of fluoride ions can also contribute to the accelerated sintering of the structure according to the authors in [6]. The fluoride ions contained in the outer layers of the amorphous NT can result in lower melting temperature of the amorphous phase and thus provide convenient sintering conditions for NT. As a result of the preferential sinteration, the NT with a smaller internal diameter can be formed.

**Figure 6:**
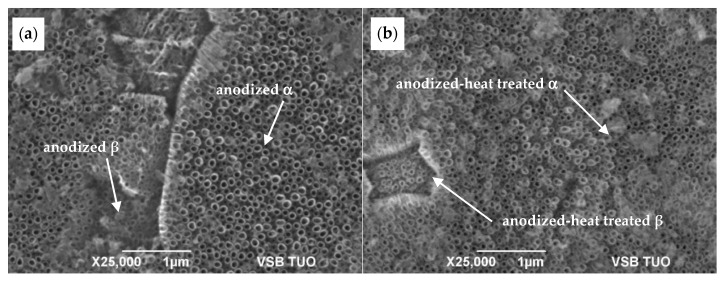
SEM SEI images of the polished sample in (**a**) as-anodized and (**b**) anodized-heat treated state.

The Table 1 seems to confirm the diameter reduction due to heat treatment for both types of surface. So, the decreasing diameter could be caused by phase transformation of amorphous NT TiO_2_ to crystalline state in the coating layer as it was confirmed by the results of XRD and Raman spectroscopy. During annealing, the density of NT changes in relation with sintering and transformation of the amorphous TiO_2_ to anatase (A) phase. Accordingly to increasing density, the volume of material decreases and NT diameter reduces to preserve integrity of tubes. Nevertheless, for more evident results other experiments have to be performed. Otherwise, this tendency could be explained by inhomogeneity of NT sizes in as-anodized coatings. Elevated temperatures, as 500 °C in this case produce thermally induced oxidation that results in further modification of the coating morphology, as it is observed in Figure 6.

### 3.2. X-ray Diffraction Analysis

**Figure 7:**
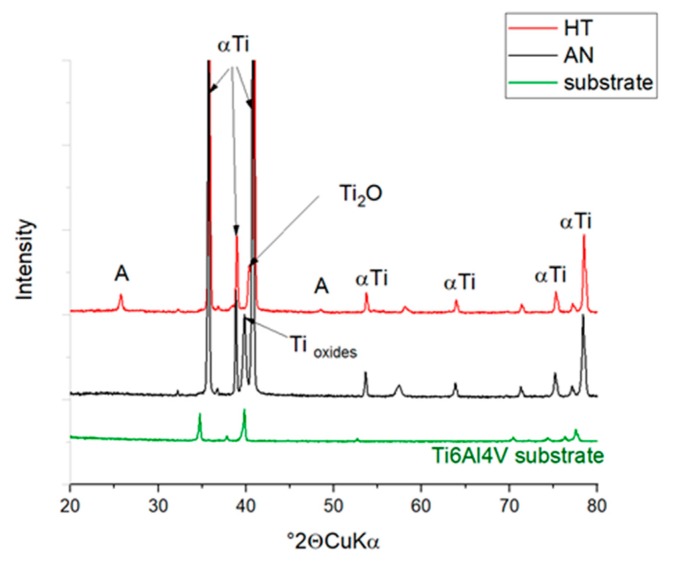
XRD patterns of the samples as-anodized (AN), anodized-heat treated (HT), and substrate. A–anatase (ICDD 01-089-4921), α Ti–alpha titanium (01-089-3725), Ti_2_O–(ICDD 01-089-4921).

The XRD analysis was selected for materials phase content confirmation. The Figure 7 shows XRD patterns of NT coatings and pattern of original substrate. The changes in phase composition are visible by appearance of new peaks.

Prior to annealing of the anodised material, the α-Ti hexagonal phase (ICDD 01-089-3725) and non-stoichiometric oxides phases such as Ti_3_O (ICDD 01-073-1583) were evaluated. Minor influence by the substrate is visible as very low intensity peaks. After heat treatment of the sample, α-Ti phase is remaining without notable changes while tetragonal anatase TiO_2_ (ICDD 01-089-4921) is observed, noted as A in Figure 7, along with hexagonal Ti_2_O (01-089-4921). The presence of Ti_2_O was reported at high heat treatment at 820 °C of TiAl alloys [17].

### 3.3. Raman Spectroscopy Analysis

**Figure 8:**
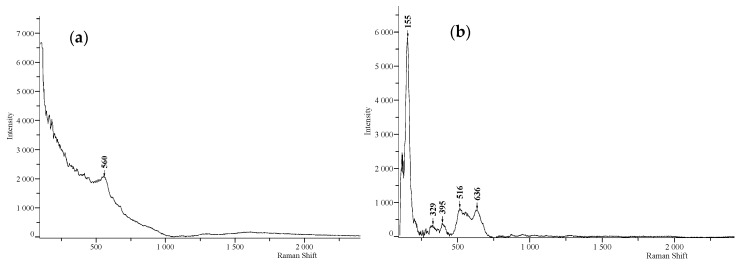
Raman spectra of the samples (**a**) as-anodized and (**b**) anodized-heat treated.

Raman spectra were recorded for as-anodized and anodized-heat treated NT layers. For the as-anodized surface, only one band was detected with the 785 nm laser, as seen in Figure 8a. The observed band probably corresponds to the oxide layer on the substrate that is in amorphous state considering its wide band width.

Figure 8b shows the bands below 1000 cm^−1^, which corresponds to the E_g_ mode (155 cm^−1^), A_1_ mode (329 cm^−1^), B_1g_ mode (395 cm^−1^), A_1g_ + B_1g_ mode (516 cm^−1^), and E_g_ mode (636 cm^−1^) of the anatase phase of TiO_2_. The E_g_ mode is mainly caused by symmetric stretching vibrations, the B_1g_ mode is caused by symmetric bending, and the A_1g_ mode is caused by antisymmetric bending vibrations of O–Ti–O in the anatase TiO_2_ structure [18,19].

### 3.4. Corrosion Tests

**Figure 9:**
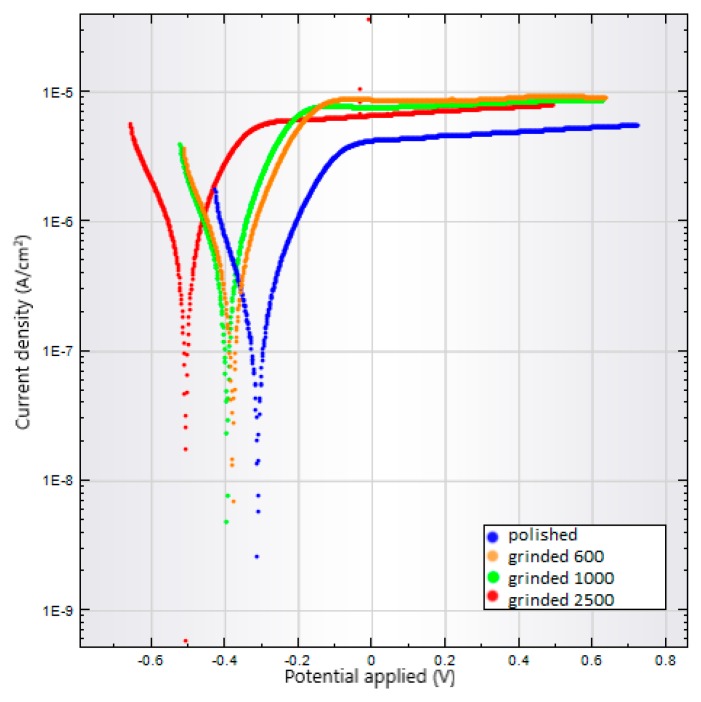
Polarization curves for the Ti6Al4V surfaces polished or ground on 600 to 2500 Grit.

**Figure 10:**
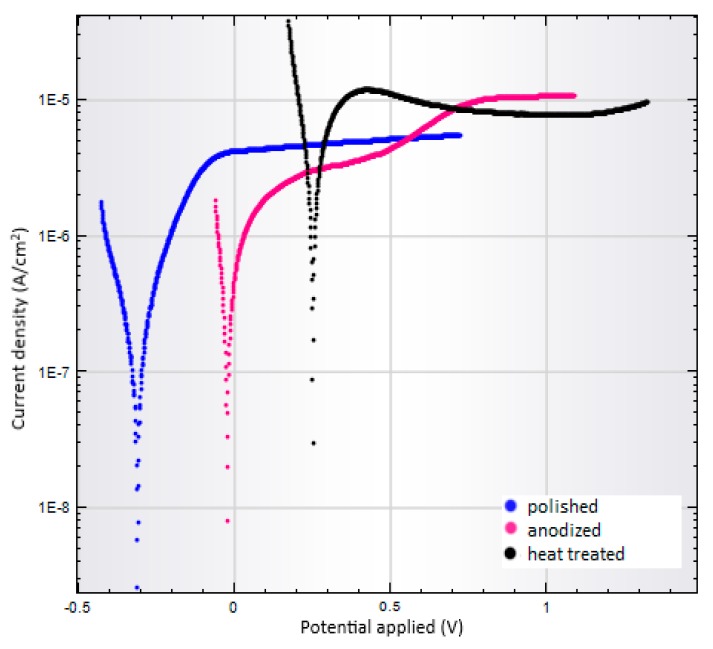
Polarization curves of a polished, as-anodized, and anodized-heat treated Ti6Al4V samples.

Corrosion tests were performed by the same manner for all prepared substrate surfaces: specimens ground on 600, 1000, and 2500 Grit, polished, as-anodized, and anodized-heat treated. Results of corrosion tests are summarized in Table 2. Comparing polarization curves are shown in Figure 9 and Figure 10.

For the first group of samples whose surfaces were finished by grinding, an increase in the corrosion rate is observed with increasing Grit number of grinding paper. The increasing rate corresponds to the decreasing corrosion potential and increasing corrosion current density. When higher Grit of the grinding papers was used, larger active surface of the substrates was produced. Conversely, the polished sample with smooth and reduced active surface shows decreased corrosion rate. The polarization curves in Figure 9 show distinct shift to more negative values of corrosion potential for ground samples comparing to the polished surface.

Unlike the polished specimen, the corrosion rate for the NT coated substrates is higher. The polarization curves in Figure 10 show a similar trend in the cathode branch for all three samples. In the anodic branch, the polished sample exhibits stable passive behavior but as-anodized surface changes the anodic branch development for a more complex one when secondary passivation occurs because of the nature of the NT layer.

For the anodized-heat treated sample, the secondary passivation effect on the anodic branch is not observed in the range of potentials measured and corrosion is the highest of all surfaces studied. The corrosion rate measured for the anodized-heat treated substrate was consistent with the positive corrosion potential, high corrosion current density, and the lowest polarization resistance (Table 2).

## 4. Conclusions

In this study the electrochemical properties of ground, polished, as-anodized, and anodized-heat treated Ti6Al4V substrates were compared. The surface of the samples studied was modified via anodic oxidation in EG electrolyte containing fluoride ions. The effect of the alloy microstructure on the growth of TiO_2_ NT was investigated and it was confirmed that the morphology of the NT coatings corresponds to the bimodal α+β microstructure of the Ti6Al4V substrates.

The phase composition of the prepared anodized layers before and after the heat treatment was evaluated using XRD and Raman spectroscopy; both methods confirmed the transformation of the amorphous NT layer into a crystalline state. The effect of the phase transformation was also observed using SEM analysis of the NT morphology, which showed the reduced diameter of the NTs after the heat treatment.

The feature of the NT layers is significantly affected by the initial surface quality and heat treatment. Based on the results of the corrosion test, it was possible to evaluate the influence of surface quality on the resulting corrosion rate. With increasing number of the paper Grit, the higher corrosion rate of the ground surfaces was measured because of the increasing active surface. The only polished samples displayed the lowest corrosion unlike the as-anodized and anodized-heat treated conditions. The effect of heat treatment on the highest corrosion rate was observed as a result of the phase transformation of the NT layer.

## Figures and Tables

**Table 1:** Dimensions of nanotubes (NT) before and after heat treatment of the Ti6Al4V substrates.

NT Dimensions Sample	Outer Diameter [nm]	Inner Diameter [nm]	Wall Thickness [nm]
Ground AN ^1^	122	82	40
Polished AN	131	84	48
Ground HT ^2^	98	62	36
Polished HT	106	56	50

Note: ^1^as-anodized; ^2^anodized-heat treated.

**Table 2:** Results of corrosion tests at 37 °C in 0.9% NaCl solution for ground, polished, as-anodized, and anodized-heat treated surfaces.

Sample of Ti6Al4V	Corrosion Rate (mm/year)	Potential Corrosion (mV)	Corrosion Current Density (nA/cm^2^)	Polarization Resistance (kΩ)
Ground 600	0.0116	−379	337	72.6
Ground 1000	0.0122	−395	352	63.6
Ground 2500	0.0219	−507	635	48.5
Polished	0.0043	−311	123	158.3
As-anodized	0.0182	−24	527	42.9
Anodized-heat treated	0.0998	251	2894	6.4
